# Non-Coding RNA Networks in ALK-Positive Anaplastic-Large Cell Lymphoma

**DOI:** 10.3390/ijms20092150

**Published:** 2019-04-30

**Authors:** Steffen Fuchs, Julian Naderi, Fabienne Meggetto

**Affiliations:** 1 Charité—Universitätsmedizin Berlin, Department of Pediatric Oncology, D-13353 Berlin, Germany; steffen.fuchs@charite.de (S.F.); julian.naderi@charite.de (J.N.); 2 Berlin Institute of Health (BIH), D-10178 Berlin, Germany; 3 German Cancer Consortium (DKTK), Partner Site Berlin, German Cancer Research Center (DKFZ), D-69120 Heidelberg, Germany; 4 Inserm, UMR1037 CRCT, F-31000 Toulouse, France; 5 Université Toulouse III-Paul Sabatier, UMR1037 CRCT, F-31000 Toulouse, France; 6 CNRS, ERL5294 CRCT, F-31000 Toulouse, France; 7 Institut Carnot Lymphome-CALYM, F-31024 Toulouse, France; 8 Laboratoire d’Excellence Toulouse Cancer-TOUCAN, F-31024 Toulouse, France

**Keywords:** non-coding RNA, competing endogenous RNA, microRNA, circRNA, lncRNA, lymphoma, anaplastic large cell lymphoma, anaplastic lymphoma kinase, drug resistance, biomarkers

## Abstract

Non-coding RNAs (ncRNAs) are essential regulators of gene expression. In recent years, it has become more and more evident that the different classes of ncRNAs, such as micro RNAs, long non-coding RNAs and circular RNAs are organized in tightly controlled networks. It has been suggested that deregulation of these networks can lead to disease. Several studies show a contribution of these so-called competing-endogenous RNA networks in various cancer entities. In this review, we highlight the involvement of ncRNA networks in anaplastic-large cell lymphoma (ALCL), a T-cell neoplasia. A majority of ALCL cases harbor the molecular hallmark of this disease, a fusion of the anaplastic lymphoma kinase (ALK) gene with the nucleophosmin (NPM, NPM1) gene leading to a permanently active kinase that promotes the malignant phenotype. We have focused especially on ncRNAs that are regulated by the *NPM*-*ALK* fusion gene and illustrate how their deregulation contributes to the pathogenesis of ALCL. Lastly, we summarize the findings and point out potential therapeutic implications.

## 1. ALK-Positive Anaplastic Large Cell Lymphoma: Pathology and Clinical Features 

Anaplastic large cell lymphoma (ALCL) is a rare and aggressive peripheral T-cell non-Hodgkin lymphoma (NHL), belonging to the group of CD30-positive lymphoproliferative disorders. The 2016 WHO classification identifies four separate biological entities: primary systemic ALK-positive ALCL (ALK-positive ALCL), primary systemic ALK-negative ALCL, primary cutaneous ALCL and breast implant-associated ALCL. Systemic ALCL accounts for approximately 3% of adult NHL and 10 to 30% of NHL in children. The ALK-positive ALCL usually affects children and young adults. The ALK-negative subtype is more commonly found in older patients over the age of 40 [1,2]. 

The first described fusion of the Anaplastic Lymphoma Kinase (ALK) gene was discovered in 1994, as a chromosomal translocation t(2;5)(p23;q35), which connects *ALK* with the *NPM* gene in ALK-positive ALCL [3]. The full functional ALK protein is a receptor-dependent tyrosine kinase [4,5]. ALK is highly expressed during the development of the nervous system. ALK activating mutations were reported in a subset of aggressive neuroblastomas, a pediatric tumor arising from precursor cells of the sympathetic nervous system [6,7]. However, the most common form of *ALK* alteration is the fusion gene formation. *NPM*-*ALK* fuses the *ALK* gene on chromosome 2 with the *NPM* gene on chromosome 5, resulting in a fusion protein with a constitutive tyrosine kinase activity. NPM is a protein that is involved in ribosome biogenesis and expressed is most cells [8]. An aberrant ALK activity enhances cell proliferation, cytoskeletal rearrangements and cellular migration through multiple intracellular signal transduction pathways, including PLCγ, PI3K-AKT, MAPK/ERK, mTOR, STAT5b and STAT3. The signal transducer and activator of transcription 3 (STAT3) has emerged as a critical mediator of NPM-ALK–induced tumorigenesis [9]. In solid tumors, *ALK* fusions were first identified in inflammatory myofibroblastic tumors, and in 2007 the echinoderm microtubule associated protein like4 (EML4)-ALK fusion protein was reported in non-small cell lung cancer (NSCLC) [10]. 

Characterized by an extranodal presentation (lung, skin and bone marrow infiltration) and male predominance, ALK-positive ALCL is highly sensitive to standard combination chemotherapy with a 5-year overall survival of approximately 70–90% in children and over 70% in adults. Treatment in adults is typically an anthracycline-based chemotherapy, such as CHOP (cyclophosphamide, doxorubicin, vincristine and prednisone) or CHOPEP (CHOP plus etoposide), as the first-line treatment. In any case, long-term toxicities and early relapses are an important clinical problem. Immunotherapy using allogeneic stem cell transplantation is a promising treatment option for adult patients that were refractory to first-line therapy. Stem cell transplantation and vinblastine maintenance are also considered reasonable options in relapsed childhood disease [2,11]. Targeted therapies for refractory ALK-positive ALCL are used as a bridging strategy prior to allogeneic transplantation. Whereas most clinical results regarding ALK inhibitors come from patients with EML4-ALK-positive NSCLC, it is clear that ALK inhibition is a potentially effective treatment strategy in all ALK-expressing malignancies evaluated so far and especially so in relapsed ALK-positive ALCL. The first reported administration of crizotinib in 7 adults with therapy-refractory ALK-positive ALCL resulted in a complete response in 3 patients and a partial response in 1 patient [11]. However, a subset of patients inevitably acquires a resistance to ALK inhibitors, including even second- (alectinib and ceritinib), or third (lorlatinib)-generation drugs used as a single therapy [12]. The results in the pediatric population are more encouraging. Indeed, the clinical trial (NCT00939770) of crizotinib in children with refractory ALK-positive ALCL, or with solid tumors, resulted in a complete response in 8 out of 9 patients with ALK-positive ALCL, exceeding the response rate in the other ALK-expressing malignancies [13]. Resistance mechanisms in ALK-positive solid tumors are caused by mutations of the *ALK* gene altering the binding of an inhibitor to the ALK protein and other signaling molecules. In the case of ALK-positive ALCL patients, who became non-responsive to crizotinib, ALK mutations in the kinase domain were observed [14]. Moreover, ALK-independent resistance mechanisms have been identified, such as the emergence of a second mutated, overexpressed, or amplified oncogene and the activation of respective downstream pathways (EGFR, KRAS, BRAF, MET, HER2, and KIT) [15]. The observed ALK inhibitor resistance clearly shows that combination therapies targeting not only ALK, but also other pro-oncogenic molecules/pathways will be required to obtain durable cures in the majority of ALK-positive ALCL patients. STAT3, the main modulator of NPM-ALK-induced tumorigenesis, is an interesting candidate. One of the different STAT3-dependent oncogenic functions is an activation of DNA-methyltransferases (DNMTs), which may lead to the epigenetic silencing of tumor suppressor genes by gene promoter methylation [16]. A role for DNMTs in the tumorigenesis of ALK-positive ALCL was recently shown by us and others. This makes a perturbation of STAT3 signaling, as well as the direct inhibition of DNMTs, potential therapeutic targets in combination with ALK-inhibition in ALCL [17,18]. Unfortunately, currently available STAT3 inhibitors have therapeutic limitations, due to their low specificity [19]. Another therapeutic possibility is brentuximab vedotin, an anti-CD30 monoclonal antibody conjugated to the antimicrotubule cytotoxic agent monomethyl auristatin, which is an option in combination with ALK-inhibition in ALCL. Several clinical publications suggest that anti-CD30 immunotoxic therapy alone, or together with chemotherapy, should benefit from a combination with an ALK inhibitor in patients with chemotherapy-refractory ALK-positive ALCL [20]. Finally, the NPM-ALK fusion protein also influences the immune system and the tumor microenvironment. As mentioned before, NPM-ALK leads to an activation of the STAT3 signaling pathway, which induces the expression of the cell-surface receptor programmed death ligand1 (PD-L1, CD274, B7-H1) on tumor cells. PD-L1 binds to its receptor PD-1 on T-cells and inhibits T-cell receptor signaling. This creates a highly immunosuppressive tumor microenvironment [9,20]. The therapeutic disruption of the PD-1–PD-L1 axis by PD-L1 targeting antibodies, such as durvalumab, has been shown to be effective in NSCLC [21]. Therefore, checkpoint inhibition may also be beneficial in ALK-positive ALCL. 

Chemotherapy is the main treatment for ALK-positive ALCLs. However, drug resistance severely limits the potency of conventional chemotherapeutic and new biological agents, which is a major barrier in the treatment of ALK-positive ALCL. Many efforts are made to identify new biomarkers to evaluate patients’ prognosis and predict the response to treatment. The ALK status is routinely evaluated in the clinic by immunohistochemistry and is an important prognostic biomarker. ALK-positive ALCL has a more favorable prognosis than ALK-negative ALCL with a long-term survival rate of almost 80% [22]. Moreover, the International Performance Index for non-Hodgkin lymphomas can also be applied to ALCL [23]. Apart from clinical parameters such as age, performance and stage, this score also includes serum lactate dehydrogenase levels as a measure of tumor activity. Other concurrent translocations, e.g., with the *MYC* proto-oncogene, are associated with a more aggressive phenotype and might be used as a negative prognostic factor [24]. Efforts were undertaken to predict relapses through the analysis of residual tumor cells after treatment. A detection of ALCL cells in bone marrow or peripheral blood is feasible by quantitative PCR and might reveal patients at risk of a disease recurrence [25]. However, such assays are not yet used routinely in the clinic. As discussed below, non-coding RNAs contribute to ALCL pathology in different ways and are often responsible for drug resistance. They can be identified as relevant therapeutic targets, but might also be promising biomarkers for the screening of therapeutic resistance of ALK-positive ALCL.

## 2. Competing Endogenous RNA Network Hypothesis

Since the final constitution of the transcriptional landscape of the mammalian genome in 2005, it has become an undisputed fact that the central dogma of molecular biology, which considers RNAs as vectors for structural information between DNA and proteins, needs to be revised [26,27]. While up to 90% of the human genome was observed to be transcriptionally active, it seemed surprising that as little as 1.4% of the transcriptome is constituted by protein-coding mRNAs [28]. Furthermore, by comparing variables, such as the genome size and the number of protein-coding genes between different species, it became apparent that the genome of more complex eukaryotes carries a significantly larger proportion of non-coding RNAs (ncRNA) [29]. Consequently, this led to the suggestion that non-coding fractions of the human transcriptome actively contribute to the complex physiology and maintenance of highly developed organisms, like vertebrates. The discovery of tRNA in 1965 as the first characterized essential ncRNA, laid the cornerstone for a broad tackle on their respective biological significance in cell physiology and in diseases [30]. Recurrent mutations, and translocations or deletions, in non-coding parts of cancer genomes, were identified as being the driving force of disease [31]. Nowadays, it is widely accepted that the majority of ncRNAs maintain cell homeostasis through the regulation of crucial housekeeping functions. Previously mentioned tRNA and ribosomal RNA, which together make up a large percentage of the non-coding transcriptome, function both as key mediators in mRNA translation. Furthermore, smaller ncRNA families, such as small nuclear RNAs (snRNA), or micro RNAs (miRNA), are involved in alternative splicing and translational regulation, respectively. 

Lately, the role of miRNAs and their physiological impact on translation has come into focus. MicroRNAs are short, usually 20–23 nt long non-coding RNAs with a high potential for translation regulation. Since the discovery of the first miRNA lin-4 in the nematode *C. elegans* in 1993, much work has been done [32]. miRNA genes can be found in all metazoans and plants, making it clear that gene regulation by these small molecules is a highly conserved mechanism [33]. Over the last two decades, an involvement of miRNAs in almost every physiological and pathophysiological aspect of life has been confirmed, which highlights the importance of these small molecules [34]. The aberrant expression of miRNAs has been identified in a variety of tumor entities. The first cancer type that was linked to miRNAs was B-cell chronic lymphocytic leukemia (B-CLL) [35]. Strikingly, miR-15 and 16 are located in a chromosomal region that is frequently deleted in B-CLL, leading to a decreased expression of these miRNAs. Further analysis showed that these miRNAs target the anti-apoptotic protein BCL-2. Therefore, a downregulation of miR-15 and 16 leads to the prolonged survival of B-CLL cells [36]. Further contribution of miRNAs to cancer pathogenesis has been demonstrated, for example, in neuroblastoma, anaplastic large cell lymphoma and almost every other entity [37,38].

So far, in the general understanding, miRNAs mostly target 3′untranslated regions (3′UTRs) of their respective mRNA targets by the complementary binding of their characteristic seed sequences, usually represented by the nucleotides 2 to 8 of the miRNA, to microRNA response elements (MREs). *In silico* predictions could demonstrate that approximately 60% of all protein-coding “classical” genes contain such MREs and also show that each miRNA could regulate hundreds of mRNAs. The subsequent activation of the microRNA-induced silencing complex (miRISC) leads to a modulation of target mRNA decay, or initiation of translation repression, dependent on the degree of sequence complementarity [39,40]. Moreover, direct interactions with other mRNA regions, such as coding sequences, 5′UTRs and even regulatory units like gene promoters, have been validated and are associated not only to translational repression, but also to activation [41].

In 2012, Salmena et al. [42] introduced a new mindset regarding transcriptional regulation by non-coding and coding RNAs in a unifying interplay of major elements of the human transcriptome, including long non-coding RNAs (lncRNA), circular RNAs (circRNA), pseudogenes, mRNAs and microRNAs, with the latter playing a central role. The proposed competing endogenous RNA (ceRNA) hypothesis states that the mentioned entities of the transcriptome perpetuate the systems’ balance through competition for a limited pool of miRNAs in each single cell. Furthermore, it is alleged that the availability of MREs as a fluctuating variable opens opportunities for an indirect crosstalk between different RNA families. In this respect, the deregulation of one active ceRNA molecule can influence the translation of RNAs carrying identical MREs by attracting statistically more or less miRNAs and therefore deregulating the repression of its competitors. This may drastically potentiate the effect of perturbation of one single ncRNA molecule. The described complexity of non-coding RNAs in a cell may get even more complicated. In a recent publication by David Bartel’s group, it was shown that in *Saccharomyces cerevisiae*, excised introns accumulate in stress conditions and potentially function as non-coding RNAs regulating cellular growth [43]. Whether introns can have a similar molecular function in more complex eukaryotes has yet to be proven. The transfer of the ncRNA network model into a representative cellular context is not trivial. Cofounding factors such as transcript abundance, miRNA:ceRNA affinity, cellular sub-localization and tissue-specific expression have first to be fully understood to reduce insignificant background noise. In fact, the optimal condition for ceRNA activity was found to be an equilibrium state of miRNA:ceRNA, which in turn diminishes the importance of highly expressed molecules due to their low chance of being sequestrated by miRNA competition [44]. The binding affinities of miRNAs to their respective ceRNA are clearly dependent on their sequence complementarity. Therefore, a higher binding affinity between the miRNA seed sequence and MRE boosts the ceRNA’s competitive strength in comparison to other low affinity targets of the same shared miRNA [45]. Thus, RNA editing as a central process of RNA maturation at the pre-mRNA level can affect ceRNA activity and sequestration in a tissue specific manner by, for example, modifying MREs through the A to I conversion by enzymes like ADAR1 [46,47]. Considering the amount of known miRNA genes and MRE harboring transcripts in the human transcriptome, this model of competing endogenous RNAs in a self-organized context adds a new layer of complexity to post-transcriptional and translational regulation mediated by microRNAs as a hub for RNA crosstalk. 

## 3. Non-Coding RNA Networks Contribute to Cancer Pathogenesis

### 3.1. Competing Endogenous RNAs are Often Deregulated in Cancer

Conveniently, recent studies demonstrate that cancer, with all its unique translocations, deletions and amplifications, constitutes a decent model for ceRNA interaction analysis [48]. Losses and amplifications of gene *loci* are frequently occurring events in various cancer entities such as neuroblastoma [49]. Consequently, abundancies of MREs carried by affected coding and non-coding sections are altered and in turn disturb the repression of miRNA competitors. As an example, Poliseno and colleagues showed that the amplifications of non-coding gene *loci* can initiate microRNA sponging by inducing an oversupply of MREs. The *KRAS1P* locus at 6p11-12 was found to be amplified in different human tumors, including neuroblastoma [49]. Pseudogenes like *KRAS1P*, compared to the functional *KRAS* mRNA, share, due to their high sequence similarity, a majority if not even all MREs with their counterparts, and thus present as ideal miRNA decoy targets. Transcriptional competition on the RNA level indicates a proto-oncogenic role of *KRAS1P* based on its ceRNA activity [50]. 

Chromosomal translocations do not directly affect MRE abundancies, the way deletions or amplifications do. Nevertheless, it has been proposed that fusion proteins, such as BCR-ABL, commonly found in chronic myelogenous leukemia, following t(9:22) translocation, or PML-RARα, following t(15:17) translocation (which typically occurs in acute promyelocytic leukemia), can contribute to a malignant phenotype due to their permanently active domains, but also by disturbing the ceRNA balances on the RNA level [51,52]. Ding et al. identified that a UTR swap of PML, now connected to the 3′UTR of *RARα* in the *PML-RARα* fusion gene, introduced *c-Myc* as a new potential ceRNA by competition for the miRNA let-7 [53]. The associated perturbation of the 3′UTR expression and subsequent translational regulation of fusion genes could provide new opportunities in the treatment of fusion gene driven cancers such as ALK-positive ALCL. 

### 3.2. Epigenetic Modifications of miRNAs Lead to Disruption of Non-Coding RNA Networks in Cancer

Epigenetic changes can be seen as modifiers of the genetic code, without changing the DNA sequence. They occur due to environmental influences, aging, cancer and other diseases. Epigenetic modifications basically involve two major events: DNA methylation and histone modifications [54]. Cytosine methylation is the most widely studied epigenetic modification. In such cases, a cytosine is methylated at position 5, which almost exclusively happens in the context of CpG-dinucleotides (shorthand for cytosine-phosphate-guanine). Such CpG-sites are mostly found in larger assemblies, called CpG-islands, and are enriched in eukaryotic promoters and regulatory elements. Therefore, they are prone to modifying the gene expression [55]. CpG-island methylation characteristically leads to gene silencing, which is mediated by Methyl-CpG-Binding Domain (MBD) proteins that maintain the silencing state due to the recruitment of histone-modifying proteins and other components [56]. It was also suggested that CpG methylation might hinder transcription factor (TF) binding and therefore lead to transcriptional silencing [57]. In cancers, the promoter regions of tumor suppressor genes and miRNAs can be hypermethylated, which leads to the inactivation of these genes. In the case of miRNA silencing, the targeted cancer-associated gene is not repressed and can lead to tumor progression [58].

The other important part of the epigenetic system is histone modification. Histones are susceptible to posttranslational modifications. Such common modifications include methylation, acetylation, phosphorylation, and can impact the activation state of chromatin [59]. For example, transcriptionally active chromatin, euchromatin, is rich in acetylated histones, such as H3K27ac (stands for histone 3 acetylated on a lysine residue at position 27), or trimethylated histones like H3K4me3 (stands for trimethylation of histone 3 at lysine 4). On the other hand, heterochromatin, which is transcriptionally silent, has a low acetylated histone content and is rich in trimethylated H3K27me3 and H3K9me3 [60]. 

Finally, these two epigenetic systems have been shown to interact with each other, e.g., by linking DNA methylation to certain histone modifications, which shows that the interplay is important for the regulation of gene expression [61]. Moreover, some miRNAs control players of the epigenetic machinery, like DNA-methyltransferases (DNMTs). As an example, Congras et al. identified miR-125b as being repressed in ALK-positive ALCL. NPM-ALK induced the activity of the DNA methyltransferase 1 (DNMT1) mediated promoter hypermethylation [62]. This highlights the complexity of the entire system and shows that the silencing of one miRNA can have far-reaching consequences [63]. 

## 4. Non-Coding RNA Networks Involved in the Biology of ALK-Positive ALCL

### 4.1. Deregulated miRNAs in the Pathogenesis of ALCL

In 2010, Merkel et al. started a pioneering investigation on the influence of microRNAs in ALCL. The aim was to identify the subtype-specific expression of functionally active miRNAs in both ALK-positive and ALK-negative ALCL to clarify physiological processes under an aberrant ALK expression, and for therapeutic and prognostic applications [64]. By using a microarray-based differential expression analysis, they first described ALK-positive and ALK-negative ALCL specific miRNA signatures from cell lines compared to a healthy T-cell pool, which were then used in several subsequent studies. The group demonstrated a significant upregulation of several miR-17-92 cluster members in ALK-positive ALCL from transgenic mouse models and primary tumor tissues. Furthermore, they recorded a strong upregulation of miR-155 in ALK-negative ALCL and a reduced expression of miR-101 in ALK-positive, as well as in ALK-negative ALCL. Strikingly, the miR-17-92 cluster was already shown to play major oncogenic roles in different cancer entities like neuroblastoma, and was thus displayed as a convenient clue for further research [65]. As a follow-up on their initial finding, in 2011, Merkel et al. showed that the reintroduction of miR-101 led to reduced proliferation in ALK-positive, but not ALK-negative cells via the inhibition of the miR-101 target mRNA mammalian target of rapamycin (mTOR). Consistent with these findings, the inhibition of mTOR strongly inhibited the proliferation of ALK-positive ALCL cells only, which pointed to a strong dependence of the ALK-positive ALCL subtype on the mTOR signaling pathway [64,66].

Later in 2014, Liu et al. reported that a high expression of miR-155 in ALK-negative ALCL was strongly anti-correlated to its promoter methylation [67]. Furthermore, treatment with miR-155 inhibitors led to reduced tumor growth in vivo, highlighting a tumor-driver function of miR-155. In parallel, a reported downregulation of miR-155 was shown to play a controlling role in ALK-positive ALCL. Mice with a miR-155 deficiency showed skewing toward Th2-differentiation along with a low expression of interferon-γ. Taken together with previously mentioned elevated levels of PD-L1, this establishes a highly immunosuppressive tumor microenvironment, characteristic of the unique ALK-positive immunophenotype [38]. Therefore, oncogenic miR-155 displays a very interesting example of microRNA-induced deregulation due to its tumor promoting or repressing functions in both ALK-negative and ALK-positive ALCL, respectively, using different oncogenic mechanisms of action. A strong connection between miRNAs and the immunophenotype of ALK-positive ALCL was already proposed in 2011. In a microarray-based approach, Matsuyama et al. revealed an NPM-ALK-STAT3-miR-135b axis, strongly polarizing T-cell differentiation toward the IL-17 producing immunophenotype through the suppression of the Th2 master regulators STAT6 and GATA3 [68], leading to the development of a pro-inflammatory tumor microenvironment. The suppression of miR-135b by an inhibitor caused a decreased tumor growth in an in vivo xenograft model, highlighting its potential tumor promoting function.

The importance of STAT3 as a major transcription factor under a strong ALK regulation should emerge as a key factor regarding miRNA regulation in ALCL. Apart from miR-135b, in 2015, in a study conducted by our group, we identified another crucial miRNA that is attenuated by the NPM-ALK dependent STAT3 expression. DNA hypermethylation of the *MIR150* gene by STAT3 dependent DNMT1 activation led to a low expression of miR-150 in ALK-positive ALCL. Therefore, the miR-150 de-repression by the DNMT1 inhibitor decitabine, led to antineoplastic activity in murine NPM-ALK-positive xenograft models, suggesting a potential application of hypomethylating drugs in tumors resistant to ALK-inhibition [17]. Pursuing this line of conduct, we recently identified another hypermethylated miRNA under NPM-ALK dependent epigenetic repression. By targeting cyclin dependent kinase 6 (CDK6), the overexpression of miR-497 in NPM-ALK-positive ALCL caused a growth inhibition and cell cycle arrest. This highlights the cell cycle regulator CDK6 as a new target for therapeutic approaches, using the CDK4/6 selective inhibitor palbocilib in ALK-positive ALCL [69]. An overview of deregulated miRNAs identified in ALCL so far is shown in Table 1. In summary, miRNAs are modulators of crucial cancer-associated signaling pathways in ALK-positive ALCL. Starting with simple expression signatures in 2010, nowadays miRNAs are proposed biomarkers for risk-stratification, and after having in part at least exploited the affected signaling pathways, they are promising agents for future treatment approaches of ALK-positive ALCL. 

### 4.2. Long Non-Coding RNAs

#### 4.2.1. Long Non-Coding RNAs have Regulatory Functions

Another important class of non-coding RNAs are long non-coding RNAs (lncRNA, or lincRNA for long-interfering RNA). They are characterized by a length of more than 200 nucleotides but can be much longer. Most lncRNAs are intergenic [73]. Large research consortia, such as FANTOM (Functional Annotation of the Mammalian Genome) from the RIKEN research institute in Japan (http://fantom.gsc.riken.jp) tried to elucidate the role of all mammalian transcripts. They identified 27,919 human lncRNA genes [74]. Interestingly, further RNA sequencing efforts, including cooperative studies from the ENCODE consortium, showed that lncRNAs have a higher tissue-specific expression than protein-coding genes [75]. In the same study Derrien et al. also revealed that a great extent of lncRNAs are non-polyadenylated and are enriched in the nucleus. This points to their role as epigenetic regulators. Indeed, several groups demonstrated that one function of nuclear lncRNAs is the recruitment of chromatin modifiers, like DNA methyltransferases or histone modifying enzymes, to genomic loci, thereby regulating chromatin accessibility. In this context, lncRNAs can form secondary structures that foster such protein interactions [76]. Many lncRNAs have been identified in the cytoplasm, where they mainly regulate the stability and translation of other transcripts by base-pairing and the recruitment of regulatory proteins. For example, it was shown that a lncRNA interacting with ALU repetitive elements in the 3′UTR of an mRNA can induce the binding of Staufen double-stranded RNA-binding protein 1 (STAU1), which subsequently leads to mRNA decay [77]. An example of positive regulation is the human β-site APP-cleaving enzyme 1 (*BACE1*) gene. Here, a protein-coding mRNA and an antisense transcript called *BACE1-AS* are transcribed from a single locus. This lncRNA binds to *BACE1*, stabilizes it and increases the protein expression. The BACE1 protein is an important enzyme in the pathophysiology of Alzheimer’s disease, and hence *BACE1-AS* actively contributes to disease progression [78]. 

Apart from these described functions, lncRNAs also play a role as competing-endogenous RNAs, as mentioned above. Irene Bozzoni’s group identified, for example, the muscle-specific RNA lnc-MD1 [79]. This lncRNA binds miR-133 and miR-135 in an inactivating manner. Thus, their targets, the transcription factors mastermind-like transcriptional coactivator 1 (MAML1) and myocyte-enhancer factor 2C (MEF2C), are no longer inhibited. Consequently, muscle-specific genes are expressed, and the cells differentiate. Moreover, lncRNAs can also be host transcripts for other ceRNAs, like miRNAs. Since miRNAs are often organized in families having similar seed sequences and regulating the same targets, it makes sense that approximately 37% of miRNAs are organized in clusters at the genomic level and are controlled by the same transcriptional unit [80]. In approximately 18% of cases, miRNAs are produced from lncRNAs and are cleaved by the microprocessor complex, which consists of the double-stranded RNA-binding protein DiGeorge syndrome critical region 8 (DGCR8) and the RNase III Drosha [81]. These enzymes play pivotal roles in canonical miRNA biogenesis. Sun et al. revealed that the lncRNA MIR100HG, which harbors miR-100, let-7a2 and miR-125b1, regulates cell-cycle progression independently of the function of the miRNAs [82]. They demonstrated that the lncRNA binds to the RNA-binding protein Hu-Antigen R (HuR) and assists its interactions. Lastly, in a recent study, Julia Salzman’s team showed that lncRNAs can also give rise to circRNAs, making the fine-tuned non-coding RNA network regulation even more complex [83].

#### 4.2.2. LncRNA—An Inconspicuous Fellow in Cancer Pathogenicity

The described functions of lncRNAs in gene regulation pinpoint an association of these molecules to diseases such as cancer when they are deregulated. Indeed, several lncRNAs have been found to have either tumor-promoting or tumor-inhibiting functions in diverse tumor entities. One of the first lncRNAs that was identified in cancer was *DD3* [84]. By using the differential display method, Bussemakers et al. showed an overexpression of the lncRNA in prostate cancer samples in comparison to normal adjacent tissue. Nowadays, *DD3* is investigated as a potential biomarker for prostate cancer in urine [85]. Another example for lncRNAs involved in cancer comes from a study of the pediatric cancer neuroblastoma [86]. This tumor entity is characterized by a high degree of chromosomal instability. One locus that is frequently deleted in high-risk neuroblastoma is 6p22.3. Two prominent transcripts of this locus are the lncRNAs *CASC15* and *NBAT1*. Mondal et al. showed that both lncRNAs act as tumor suppressors and are involved in the differentiation of neuroblasts. These two studies are only representative examples of the plethora of lncRNAs identified in cancer. For a more comprehensive overview, we recommend the recent review by Maite Huarte [87].

#### 4.2.3. LncRNAs in ALK-Positive ALCL

So far, only two reports exist identifying lncRNAs in ALCL. Kwang-Huei Lin’s group studied lncRNAs in ALK-positive ALCL by comparing the expression of matched tumor samples with normal tissue samples through a lncRNA-microarray [88]. They detected 51 differentially expressed candidate transcripts. Among them, *LINC01013* was upregulated. A depletion of the lncRNA in KARPAS-299 ALK-positive ALCL cells reduced invasion. Interestingly, they also pinpointed a putative mechanism of the *LINC01013* function, which is an induction of the transcription factor snail family transcriptional repressor 1 (Snail). Snail is a crucial player in embryonic development and important for mesoderm formation. It downregulates ectodermal genes within the mesoderm and induces a migratory phenotype. Importantly, Snail has also been shown to be involved in breast cancer recurrence by the induction of the epithelial-to-mesenchymal transition (EMT), which leads to a more invasive phenotype of the cancer cells [89]. This finding highlights the oncogenic role of *LINC01013*. In a follow-up study from the same group, they compared the lncRNA expression in ALK-positive and ALK-negative cell lines and found that the lncRNA *MIR503HG* was significantly higher expressed in ALK-negative cells [90]. Huang et al. showed that upon the depletion of *MIR503HG*, the tumor growth was inhibited in vivo in a subcutaneous mouse xenograft model. Of note, it was previously shown that *MIR503HG* is the host gene of miR-503 and that the two transcripts are co-expressed [91]. They established *MIR503HG*-overexpressing SR-786 ALK-positive ALCL cells, which showed an enhanced cell proliferation in comparison to the controls. As a putative mode of action, they demonstrated that *MIR503HG* expression leads, through the upregulation of miR-503, to an inhibition of its target SMAD specific E3 ubiquitin protein ligase2 (Smurf2). Consequently, Smurf2 can now no longer ubiquitinate the transforming growth factor beta receptor1 (TGFBR1), which is a positive regulator of cell proliferation in the context of lymphoma cells. This study shows how a lncRNA functions by controlling a miRNA. As a summary, these findings illustrate how competing-endogenous RNAs influence each other and how the disruption of this tight regulation can contribute to cancer. 

### 4.3. Circular RNAs Represent a New Level of Gene Expression Regulation

#### 4.3.1. General Features of circRNAs

Circular RNA (circRNA), a not entirely novel branch of non-coding RNA, is currently undergoing a renaissance and is increasingly becoming a focus of investigators from cancer research to biotechnology [92]. Generally thought of as byproducts of canonical splicing, and therefore neglected, circRNAs are starting to reveal some of their distinct cellular functions [93]. With the increasing number of next generation sequencing studies, such as Julia Salzman’s study in 2012, it is becoming increasingly clear that circRNAs have crucial regulatory functions in differentiation, tissue homeostasis and cancer development [94,95]. CircRNAs are non-canonical ring-like transcripts ubiquitously expressed in the transcriptome of almost all eukaryotes that are known today [96]. Though the biological relevance of most circRNAs has not yet been discovered, the process of their biogenesis has started to be investigated in the recent years. A “backsplicing” reaction circularizes the pre-mRNA of the host gene. Long exon flanking intronic regions with inverted sequences and ALU-repeats facilitate this alternative splicing process by bringing splice sites into closer proximity [97]. With the 5′-end ligated to the 3′-end of the same molecule, circRNAs show an exclusive feature, making them not only unique regarding their sequence, but rendering new physiological functions possible. The absence of a freely accessible 3′-end due to the circularization results in a high stability toward RNA degrading enzymes, which leads to an extended half-life of circRNAs compared to their cognate linear counterpart. As the tissue specific expression levels of the circular and linear transcripts of the same host gene do not seem to correlate, it can be hypothesized that circular RNAs are the product of a differentially regulated alternative splicing process [97]. Recent studies indicate diverse cellular functions of members of the circRNA family in physiological and pathological processes. It has been shown that circRNAs can interfere in the gene expression regulation by acting as decoy targets for miRNAs [98]. As already mentioned, circRNAs are highly suspected of exerting ceRNA activity in a tissue specific manner [99]. Shao et al. reported that circRNAs are specifically enriched in brain tissue, as in neuropils and in dendrites, and seem to have the potential to regulate the synaptic function, differentiation of neural progenitor cells and neural plasticity [99,100]. Considering the extended half-life of circRNAs, their role as powerful translational regulators with ceRNA activity should not be underrated. Recently, Benjamin Kleaveland and colleagues were able to reveal a ncRNA interaction network in neuronal tissue centered *inter alia* around the circular RNA CDR1-AS. Its direct interactions with the lncRNA Cyrano, miR-7 and miR-671 led to the regulation of neuronal activity through ceRNA interactions [101].

Furthermore, circRNAs seem to be related to cancer development in various ways, for example through either their interaction with tumor associated RNA binding proteins or through their ability to encode regulatory peptides involved in tumor proliferation [102,103]. These findings raise important questions regarding the role of circular RNAs in tumor development and the regulation of tumor survival and proliferation.

#### 4.3.2. circRNAs as Possible Mediators of Cancer Pathogenicity

In 2011, by finding a circularized transcript originating from the antisense strand of the human *CDR-1* gene, Hansen et al. identified the probably best known and characterized circular RNA today, CDR1-AS. CDR1-AS was widely expressed in glioblastoma, while miR-7 expression was reduced. It turned out that CDR1-AS, carrying over 60 highly enriched miR-7 specific MREs and having a structurally related prolonged half-life, is a powerful ceRNA, regulating miR-7 mediated mRNA expression in glioblastoma. Furthermore, CDR1-AS showed consistent expression in other cancer types such as neuroblastoma and astrocytoma [104]. In this study, Liu et al. were able to determine associated molecular pathways directly affected by CDR1-AS ceRNA activity. Epidermal growth factor receptor (EGFR) signaling is a key pathway for cell proliferation, migration and invasion in cancer in general [105]. As it turned out, Phosphoinositide-3 kinase (PI3K) and Raf-1 proto oncogene (Raf-1), both direct targets of EGFR, and first links in PI3K/AKT and Raf/MEK/ERK signaling pathways respectively, were targets of miR-7 and therefore in direct competition with CDR1-AS [106]. Thus, CDR1-AS is directly associated with glioblastoma aggressiveness. Besides their function as competing-endogenous RNAs, circRNAs pursue tumor driving processes by the direct interaction with RNA-binding proteins (RBPs). As an example, Chen et al. discovered the association of a circular transcript of argonaute 2 (*AGO2*) and the progression of various tumor entities like colorectal cancer and neuroblastoma [102]. Under physiological conditions, AGO2, a central part of the RISC complex, actively regulates miRNA mediated mRNA decay or translational repression. However, the group identified circAGO2 as a mediator of the AGO2-miRNA interaction, thus exhibiting in parallel to its physiological function a role in translational regulation. A direct interaction of circAGO2 with the RBP Hu-antigen R (HuR) causes HuR binding and enrichment in the target mRNA 3′UTRs. This subsequently leads to a reduced AGO2 affinity, the repression of miRNA mediated suppression of the affected downstream targets and thereby the promotion of proliferation, invasion and metastasis formation. Recently, Vo et al. built up the so far most comprehensive cancer specific circRNA database (MiOncoCirc). In this study, they first showed evidence of specific circRNA expression in different cancer entities, and furthermore reported a novel class of circRNAs, so called read-through circRNAs (rt-circRNA). These consist of exons originating from adjacent genes of the same strand [107]. Importantly, it was recently shown that cancer-associated chromosomal translocations, for example the *PML/RARα* translocation in acute promyelocytic leukemia, or the *EML4/ALK* translocation in lung cancer, give rise to so-called fusion circRNAs (f-circRNAs) [108]. Interestingly, Vo et al. found that a subset of rt-circRNAs was differentially expressed in distinct cancer types. Among them was e.g., rt-circRNA RB1-ITM2B, being composed of exon 2 of the well-known tumor suppressor gene *RB1*, ligated to exon 3 of the upstream *ITM2B* gene. With mostly unacquainted functions, rt-circRNAs and f-circRNAs are an interesting group of circular RNAs, especially with regard to their commitment to MRE availabilities in ceRNA networks and their coding potential for fusion proteins in a cancer specific manner. In summary, circRNAs, despite their recent rediscovery, have been shown to have a major potential for gene regulation by using various mechanisms of direct interaction with miRNAs or RBPs, and thus they are key players in the pathogenicity of different tumor entities.

#### 4.3.3. circRNAs in ALCL

Surprisingly, very little is known about the role of circRNAs in ALCL. While most studies, as already mentioned, focused on classical miRNA:mRNA interactions, circRNAs were not considered further in the context of ALCL. As a first attempt in this direction, in 2018, Babin et al. used ALK-positive ALCL as a model system for investigations regarding the role of f-circRNAs in cancer. Focusing on the frequently occurring *NPM-ALK* fusion gene, they found that a CRISPR/Cas9-induced *NPM-ALK* translocation in mice, besides inducing the anticipated activation of oncogenic STAT3 and MEK/ERK pathways, also induced the production of novel fusion circRNAs transcribed from the generated *NPM-ALK* breakpoint (f-circNPM1-ALK) [109]. Interestingly, the f-circRNAs generated in mice were already described in human tumor-associated cells. Additionally, in 2018, Shuangyan et al. reported that f-circEA2a, originating from a distinct *EML4-ALK* translocation, induced migration and invasion in non-small-cell lung cancer cells [110], suggesting a biological contribution of ALK-breakpoint derived fusion circRNAs to the tumor phenotype. Needless to say, the role of circRNAs in ALCL has clearly been underappreciated. These pioneering studies are hopefully only the beginning of broader investigations focusing on the molecular functions of circRNAs in ALK-positive ALCL. 

### 4.4. Other ncRNA Classes

Besides the major ncRNA classes mentioned so far, a plethora of other ncRNA molecules has been identified. For the class of small RNAs in particular, recent research has revealed many subclasses, such as miRNAs, piwi-interacting RNAs (piRNA), endogenous small-interfering RNAs (siRNA), small nucleolar RNAs (snoRNA), vault RNAs (vtRNAs) and others. They have important physiological cellular functions, such as, for example, chromatin-modification, pre-mRNA processing and splicing, ribosomal RNA maturation and regulation, and modifications of other transcripts. A comprehensive review on different ncRNA classes has recently been published [111]. However, their exact functions have yet to be elucidated and their association with benign and malign diseases has just begun. In the case of ALCL, only one study was identified analyzing other non-coding RNA classes. Our group profiled snoRNAs to identify new prognostic markers in peripheral T-cell lymphoma, including ALCL [112]. SnoRNAs are highly conserved RNAs that arise from excised introns and have a function in the chemical modification of other RNAs, especially ribosomal RNA [113]. Using high-throughput quantitative real-time PCR to analyze 80 different snoRNAs, Valleron et al. found a global downregulation of snoRNAs in cancer cells, whereas a subset of 30 snoRNAs specifically characterized ALCL. Importantly, the expression level of a single snoRNA, U3 snoRNA, could differ between ALK-positive and ALK-negative ALCL. This has potential diagnostic implications for ALCL. However, given the fact that many of these ncRNA classes have just started to be investigated systematically, more studies will follow to explore their function in cancer and eventually in ALCL. 

## 5. Conclusions and Translational Aspects

**Figure 1:**
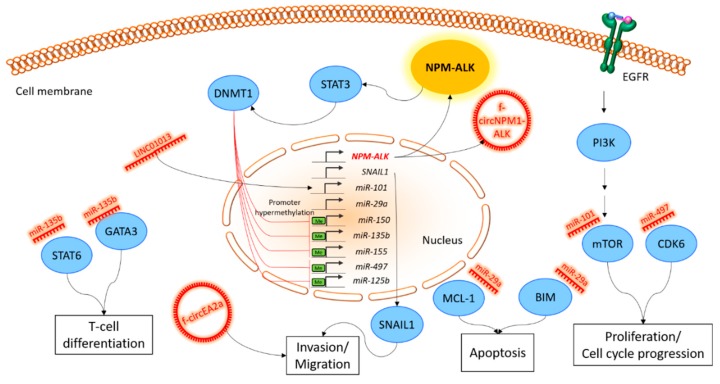
Schematic overview of non-coding RNA involvement in key cellular pathways of ALK-positive ALCL. The figure shows representative examples of the discussed ncRNAs, together with their influenced signaling pathways or the cellular function in ALCL. The expression of the NPM-ALK fusion protein as a central molecular hallmark of ALK-positive ALCL influences various pathways, including STAT3. STAT3 activates DNMT1 and subsequently methylates several miRNA promoters. The deregulation of miRNA expression strongly influences crucial signaling cascades, like PI3K/AKT/mTOR, as well as cellular hallmark processes such as proliferation, invasion and T-cell differentiation. The ceRNA activity of other non-coding RNA entities, such as circRNAs, which can arise from gene fusion events, such as *NPM*-*ALK*, and the activity of lncRNAs, are dependent on a defined pool of miRNAs and are thereby regulated by the NPM-ALK activity. STAT3, signal transducer and activator of transcription 3; DNMT1, DNA (cytosine-5)-methyltransferase 1; GATA3, GATA binding protein 3; STAT6, signal transducer and activator of transcription 6; SNAIL1, snail family zinc finger 1; MCL-1, myeloid cell leukemia 1; BIM, Bcl-2-like protein 11; mTOR, mammalian target of rapamycin; CDK6, cyclin dependent kinase 6; PI3K, phosphoinositide 3-kinase; EGFR, epidermal growth factor receptor; NPM, nucleophosmin; EML4, echinoderm microtubule associated protein like 4; ALK, anaplastic lymphoma kinase.

Until recently, the development of cancer was predominantly explained by mutations of protein-encoding genes. However, it has become more and more clear that cancer, as well as ALCL, is a multifactorial disease [114]. A new level of complexity emerged after the identification of miRNAs in the non-coding genome and the insight that they are important players in gene expression regulation and cancer biology [115]. To make the situation even more complicated, in recent years many other ncRNA classes were identified that were found to be organized in tightly controlled networks. In the above-mentioned examples, we illustrate how the disruption of these players may lead to diseases and contribute in particular to ALK-positive ALCL pathogenesis. The discussed non-coding RNAs in their intracellular context in ALCL are summarized in Figure 1. 

We explain that deregulation often takes place by epigenetic silencing. Such epigenetic modifications, especially DNA methylation by DNA-methyltransferases, or repressive histone modifications, are potent mechanisms for gene expression control. They are necessary to install a cell’s tissue-specific phenotype, but tend to be abused by cancer cells to get rid of “obstacles”, such as tumor suppressor genes or miRNAs. The identified involvement of ncRNA networks in cancer and ALCL has the potential to use those molecules as biomarkers and therapeutic targets. ALK-positive ALCL is often resistant to the standard chemotherapy regimen consisting of doxorubicin. As mentioned before, we showed that treatment with doxorubicin inhibits DNA-methyltransferase 1, which is normally responsible for the silencing of miR-125 through promoter hypermethylation. The restored miR-125 expression increased the survival of cancer cells and correlated with the early relapse of patients. This indicates a possible pharmaco-resistance mechanism to treatment with doxorubicin mediated by miR-125. Therefore, we proposed miR-125b as a potential theranostic tool with potential clinical applications for doxorubicin-containing polychemotherapy (European patent EP16306209). On the other hand, ncRNAs often regulate many different transcripts, and this potentiates their function. It makes their use as therapeutic molecules tempting. However, the instability of linear RNA, poor pharmacokinetics and low potency, have prevented their clinical application so far. Due to their covalently closed circular structure, the more stable circular RNAs could overcome these obstacles. Therefore, in the coming years, one of the main tasks of RNA biologists will be to translate their fundamental findings of ncRNA biology in both cancer and ALCL into clinically applicable strategies from which patients will finally benefit. 

## Figures and Tables

**Table 1:** Overview of miRNAs in ALCL. The previously discussed miRNAs with affected pathways are mentioned, along with whether they have a tumor suppressive (TS) or oncogenic (OncomiR) function in ALK-positive ALCL. PI3K, phosphoinositide 3-kinase; AKT, RAC-alpha serine/threonine-protein kinase; mTOR, mammalian target of rapamycin; TLR, toll-like receptor; STAT3, signal transducer and activator of transcription 3; MCL-1, myeloid cell leukemia; VEGF, vascular endothelial growth factor; STAT6, signal transducer and activator of transcription 6; GATA3, GATA binding protein 3; TCR, T-cell receptor; NF-kappaB, nuclear factor ‘kappa-light-chain-enhancer’ of activated B-cells; CDK6, cyclin-dependent kinase 6; DNA, desoxyribonucleic acid.

miRNA		Associated Pathway/Genes	References
Mir-101	TS miR	PI3K/AKT/mTOR signaling	[64,66]
Mir-155	TS miR	TLR signaling	[38,64,67]
Mir-17-92	OncomiR	STAT3 signaling	[64,67,70]
Mir-29a	TS miR	Intrinsic apoptosis (MCL-1)	[71]
Mir-16	TS miR	VEGF pathway (VEGF)	[72]
Mir-135b	OncomiR	Th2 polarization (STAT6, GATA3)	[68]
Mir-181a	TS miR	T-cell differentiation, TCR signaling	[38]
Mir-146a	TS miR	NF-kappaB pathway	[38,67]
Mir-150	TS miR	T-cell differentiation, TCR signaling	[17,38]
Mir-497	TS miR	Cell cycle (CDK6)	[69]
Mir-125b	TS miR	DNA hypermethylation	[62]

## Abbreviations

circRNA	Circular RNA
miRNA	Micro RNA
ceRNA	Competing endogenous RNA
ALCL	Anaplastic large cell lymphoma
miRNA	Micro RNA
lncRNA	Long non-coding RNA
FANTOM	Functional Annotation of the Mammalian Genome
ENCODE	Encyclopedia of DNA Elements
PI3K	Phosphoinositide 3-kinase
AKT	Protein kinase B
mTOR	Mammalian target of rapamycin
TLR	Toll-like receptor
STAT3	Signal inducer and activator of transcription 3
STAT6	Signal inducer and activator of transcription 6
MCL-1	Myeloid cell leukemia 1
GATA6	GATA-binding factor 6
VEGF	Vascular endothelial growth factor
TCR	T-cell receptor
CDK6	Cyclin-dependent kinase 6
NPM	Nucleophosmin
ALK	Anaplastic lymphoma kinase
PML	Promyelocytic leukemia protein
RARα	Retinoic acid receptor alpha
MRE	Micro RNA response element
DNMT1	DNA methyltransferase 1
LncRNA	Long non-coding RNA
